# Associations between Blood Glucose and Carotid Intima-Media Thickness Disappear after Adjustment for Shared Risk Factors: The KORA F4 Study

**DOI:** 10.1371/journal.pone.0052590

**Published:** 2012-12-21

**Authors:** Bernd Kowall, Nina Ebert, Cornelia Then, Joachim Thiery, Wolfgang Koenig, Christa Meisinger, Wolfgang Rathmann, Jochen Seissler

**Affiliations:** 1 Institute of Biometrics and Epidemiology, German Diabetes Center, Leibniz Center for Diabetes Research at Heinrich Heine University Düsseldorf, Düsseldorf, Germany; 2 Diabetes Zentrum, Medizinische Klinik und Poliklinik IV - Campus Innenstadt, Klinikum der Ludwig-Maximilians-Universität, München, Germany; 3 Institute of Laboratory Medicine, Clinical Chemistry and Molecular Diagnostics, University of Leipzig, Leipzig, Germany; 4 Department of Internal Medicine II – Cardiology, University of Ulm, Medical Center, Ulm, Germany; 5 Helmholtz Zentrum München, German Research Center for Environmental Health, Institute of Epidemiology II, Neuherberg, Germany; 6 Clinical Cooperation Group Diabetes, Ludwig-Maximilians-Universität München and Helmholtz Zentrum München, München, Germany; INRCA, Italy

## Abstract

**Objective:**

The association between blood glucose and carotid intima-media thickness (CIMT) is considered to be established knowledge. We aimed to assess whether associations between different measures of glycaemia and CIMT are actually independent of anthropometric variables and metabolic risk factors. Moreover, we checked published studies for the adjustment for shared risk factors of blood glucose and CIMT.

**Methods:**

Fasting glucose, 2-hour glucose, HbA1c, and CIMT were measured in 31-81-years-old participants of the population-based Cooperative Health Research in the Region of Augsburg (KORA) F4 study in Southern Germany (n = 2,663). CIMT was assessed according to the Rotterdam protocol. Linear and logistic regression models with adjustment for age, sex, anthropometric measures, hypertension, and dyslipidaemia were fitted to assess the association between continuous measures of glycaemia, and categories of glucose regulation, respectively, with CIMT.

**Results:**

We found a 0.10 mm increase (95%-confidence interval: 0.08–0.12) in CIMT in subjects with compared to subjects without diabetes in crude analysis. This increase was not significant in age-sex adjusted models (p = 0.17). Likewise, neither impaired fasting glucose (p = 0.22) nor impaired glucose tolerance (p = 0.93) were associated with CIMT after adjustment for age, sex, and waist circumference. In multivariable adjusted models, age, sex, hypertension, waist circumference, HDL and LDL cholesterol, but neither fasting glucose nor 2-hour glucose nor HbA1c were associated with elevated CIMT. Literature findings are inconclusive regarding an independent association of glucose levels and CIMT.

**Conclusion:**

CIMT is highly dependent on traditional cardiovascular risk factors, but no relationships between blood glucose and CIMT were found after adjustment for age, sex, and anthropometric variables.

## Introduction

The association between blood glucose and carotid intima-media thickness (CIMT) is considered to be established knowledge as can be seen from reviews and meta-analyses [1]–[4]. Moreover, such an association seems to be plausible because both hyperglycaemia and CIMT predict cardiovascular outcomes: CIMT is accepted as a marker of early atherosclerosis [5]–[7], Type 2 diabetes is related to an increased risk of atherosclerotic diseases [8], [9], and an increase in cardiovascular risk was observed in elevated glucose levels below the diabetic range [10], [11].

However, a closer look at the meta-analyses reveals that there are still open questions. Components of the metabolic syndrome do not only predict hyperglycaemia, but also the thickening of arterial walls [12]–[14]. Therefore, associations between hyperglycaemia and CIMT should be carefully adjusted for obesity, hyperlipidaemia, and hypertension. In a meta-analysis of 11 studies, a significant correlation between postprandial glucose and CIMT was found [2]. However, this correlation was not adjusted for age, sex, or other covariables. In another meta-analysis, a difference of 0.13 mm in CIMT was reported between diabetic and non-diabetic subjects without adjustment for covariables [3]. In a further meta-analysis of nine studies with adjustment for age, but for no other covariables, Brohall et al. found that CIMT was slightly larger in impaired glucose tolerance (IGT) than in normal glucose tolerance (NGT) [4]. In their own cohort study of 64-year-old women, however, the authors did not find an association between IGT and CIMT after matching for BMI and waist-to-hip ratio [4].

Only few authors attached importance to careful adjustment for metabolic risk factors fitting several regression models with different sets of CVD risk factors and anthropometric variables [15]–[18]. Thus, the objective of the present study is to assess the association between blood glucose and CIMT in a population-based study. To this purpose, we will use a variety of continuous and categorical glucose measures (fasting plasma glucose (FPG), 2-hour plasma glucose (2hPG), HbA1c; categories of glucose regulation according to the WHO classification [19]), and we will systematically examine how the association between glucose and CIMT depends on adjustment for age, sex, and CVD risk factors like BMI, waist circumference, HDL and LDL cholesterol levels, and hypertension. Furthermore, we will check whether literature findings are conclusive regarding an independent association of glucose levels and CIMT.

## Methods

### Study Population

The KORA S4 study is a population-based health survey conducted in the city of Augsburg and two surrounding counties between 1999 and 2001. Briefly, a total sample of 6640 subjects was drawn in a two-stage cluster sample from the target population consisting of all German residents of the region aged 25 to 74 years. Of the randomly selected 6640 subjects, 4261 (64.2%) participated in the S4 baseline study. KORA F4 is a follow-up examination carried out 7 years after the baseline S4; altogether 3080 persons participated in KORA F4. Loss to follow-up was due to subjects who had died in the meantime (n = 176, 4%), lived too far outside the study region or were completely lost to follow-up (n = 206, 5%), or had demanded deletion of their address data (n = 12, 0.2%). Of the remaining 3867 eligible persons, 174 could not be contacted, 218 were unable to come because they were too ill or had no time, and 395 were not willing to participate in this follow-up, giving a response rate of 79.6%.

Of the 3080 participants in KORA F4, 417 were excluded because they did not have measurements of CIMT, or because they did not have previously known diabetes, and did not undergo complete oral glucose tolerance tests (OGTT).

### Ethics Statement

The investigations were carried out in accordance with the Declaration of Helsinki, including written informed consent of all participants. All study methods were approved by the ethics committee of the Bavarian Chamber of Physicians, Munich.

### Ascertainment of Diabetes and Prediabetes

Self-reported diabetes cases and the date of diagnoses were validated by contacting the general practitioners, who treated the participants. Among all subjects without diabetes, OGTT were performed during the morning hours (range 7∶00 to 11∶00 h). Participants were asked to fast for at least 10 hours overnight, to avoid heavy physical activity on the day before examination, and to refrain from smoking before and during the test. Exclusion criteria for the OGTT were known diabetes or diabetes treatment, and acute illnesses (infection, fever, acute gastrointestinal diseases). Fasting venous blood glucose was sampled and 75 g of anhydrous glucose were given (Dextro OGT, Boehringer Mannheim).

Diabetes was defined by FPG ≥7.0 mmol/l and/or 2 h-glucose ≥11.1 mmol/l. Prediabetes was divided into isolated impaired fasting glucose (i-IFG), isolated impaired glucose tolerance (i-IGT), and combined IFG and IGT (IFG/IGT). I-IFG was defined as FPG ≥6.1, <7.0 mmol/l and 2 h-glucose <7.8 mmol/l. I-IGT was defined as 2 h-glucose ≥7.8, <11.1 mmol/l and FPG <6.1 mmol/l [19].

### Measurement of IMT

Two certified investigators examined extracranial carotid arteries bilateraly with a B-mode ultrasound using 10-MHz linear array transducer and a high resolution instrument (Sonoline G, Siemens Medical Solutions, Munich, Germany). Ultrasound protocol and measurements were performed according to the protocol of the Rotterdam study [20]. Every participant had to lie in exactly the same position following the standardized protocol and the entire examination was continuously documented in sound and vision on DVD. After a search for the optimal longitudinal image for the far wall of the common carotid artery (CCA) CCA was evaluated at 60, 90, 120, 150 and 180 degrees marked on the Meijeŕs Arc (right carotid artery), and at 300, 270, 240, 210 and 180 degrees (left carotid artery) and recorded on a DVD videotape (BD-X20 1M, Firma JVC). Vertical and horizontal calibration measurements were performed once every week (about every 100th measurement) using an ultrasound phantom (RMI 40466, Gammex).

Measurements of intima-media-thickness (IMT) were performed off-line. Optimal scans from selected angles on both sides were frozen on the R-wave of the ECG. Carotid IMT of the far wall was determined over a length of 10 mm beginning at 0–5 mm of the dilatation of the distal CCA using an automated edge detection reading system (Prowin software, Medical Technologies International, USA). This method has been previously described in detail (Bots 1994), and permits the determination of mean (average of each of the frozen images) values for the IMT on the left and right CCA. We used the average of the measurements of 3 frozen images from both the left and right CCA to calculate artery thickness of the distal CCA ((mean left+mean right)/2). An average variable of left and right CIMT was calculated as dependent variable for regression models.

One certified reader measured all IMT scans. Reproducibility studies for IMT measurements have been performed between paired measurements of sonographers and a second reader. Measurements of intersonographer (n = 30 IMT measurements) and interreader variations (n = 50 IMT measurements) revealed coefficients of variations of 1.9% and 3.0% with Spearman correlation coefficients of ≥0.89.

### Anthropometric and Laboratory Measurements

Height, weight, waist circumference and blood pressure were measured based on standardized protocols as described previously in detail [21]. Body mass index (BMI) was calculated as weight in kilograms divided by height squared (in square meters). Hypertension was defined as blood pressure of 140/90 mm Hg or higher, or use of antihypertensive medication given that the subjects were aware of being hypertensive. Blood was collected without stasis. After blood-withdrawal the blood samples were centrifuged and kept cool (4°C) until analysis of blood glucose in the central laboratory, which took place within a maximum of 6 h after withdrawal. Blood glucose levels were assessed using the hexokinase method (Glu-Flex, Dade Behring, Marburg, Germany). Total cholesterol concentrations were measured according to enzymatic methods (Chol Flex, Dade Behring, Marburg, Germany). HbA1c was measured using the high performance liquid chromatography method (Menarini HA-8160).

### Statistical Analyses

Baseline characteristics of the study group were calculated according to categories of glucose regulation. In linear regression models, categorical variables of glucose regulation as well as continuous measures of glycaemia (FPG, 2hPG, HbA1c) were used to assess associations between glycaemia and CIMT. The linear regression models were adjusted for age and sex, and, in addition, for various components of the metabolic syndrome. To fulfill the assumptions of normally distributed residuals and of homoscedasticity, ln(CIMT) was used as dependent variable in the linear regression models. Instead of the regression coefficient ß, e^ß^ is presented which can be interpreted as the factor by which CIMT is increased per unit of the predictor variable (or, in case of categorical variables, as the factor by which CIMT is larger in a given category compared to the category of reference). Logistic regression models were conducted to predict elevated CIMT defined as CIMT values ≥75^th^ percentile. Age, sex, hypertension, HDL and LDL cholesterol, waist circumference, and one of three measures of glycaemia (FPG, 2hPG, HbA1c) were included in the logistic models.

Several additional analyses were done: stratified analyses for men and women; confinement of analyses to subjects ≥50 years; separate analyses for subjects with NGT or T2DM alone; exclusion of subjects with former myocardial infarction or stroke.

The level of statistical significance was 5%. All analyses were performed using the SAS version 9.2 (SAS institute, Cary, NC, USA).

## Results

In the study group, 189 (7.1%) persons had previously known diabetes, 99 (3.7%) cases of diabetes were newly detected, and 437 (16.4%) persons had prediabetes (Table 1). As expected, metabolic profiles were most favourable in subjects with NGT, and least advantageous in subjects with diabetes or combined IFG/IGT.

**Table 1:** Characteristics of the study group by categories of glucose regulation^a^.

	NGT	i-IFG	i-IGT	IFG+IGT	Previously undetected T2DM	Previously known T2DM
N	1938	108	271	58	99	189
Sex (male) (%)	45.6	65.7	47.2	56.9	57.6	58.7
Age (years)	52.7±12.5	61.0±10.6	63.1±11.2	64.8±8.8	65.1±10.3	67.2±9.0
BMI (kg/m^2^)	26.7±4.3	29.9±4.7	29.5±4.6	31.1±5.2	30.9±4.3	31.2±5.4
WC (cm)	90.7±12.9	102.5±12.7	98.7±14.0	105.0±14.0	104.2±11.5	105.1±12.5
HT (yes) (%)^b^	27.5	53.7	52.8	70.7	76.8	78.3
HDL (mmol/l)	1.49±0.37	1.32±0.35	1.43±0.37	1.30±0.32	1.25±0.33	1.28±0.29
LDL (mmol/l)	3.49±0.88	3.74±0.94	3.76±0.94	3.77±0.80	3.56±0.92	3.27±0.90
Triglycerides (mmol/l)	1.07 (0.75, 1.54)	1.48 (1.15, 2.16)	1.37 (1.02, 1.94)	1.93 (1.33, 2.69)	1.65 (1.17, 2.62)	1.57 (1.14, 2.27)
HbA1c (mmol/mol)	35±3	39±4	38±4	41±4	44±10	52±12
HbA1c [%]	5.4±0.3	5.7±0.3	5.6±0.3	5.9±0.3	6.2±0.9	6.9±1.1
FPG (mmol/l)	5.1±0.4	6.3±0.2	5.4±0.4	6.5±0.2	6.8±1.4	7.9±2.0
2hPG (mmol/l)	5.4±1.2	6.1±0.9	8.9±0.9	9.3±1.0	12.0±3.2	–
IMT^c^ (mm)	0.83±0.13	0.91±0.13	0.91±0.14	0.91±0.14	0.93±0.14	0.94±0.13

NGT: normal glucose tolerance; IFG: impaired fasting glucose; IGT: impaired glucose tolerance; i-IFG: isolated impaired fasting glucose; i-IGT: isolated impaired glucose tolerance; T2DM: type 2 diabetes; BMI: body mass index; WC: waist circumference; HT: hypertension; HDL: high density lipoprotein cholesterol; LDL: low density lipoprotein cholesterol; FPG: fasting plasma glucose; 2hPG: 2-hour plasma glucose; IMT: intima media thickness.

a mean ± standard deviation, median (first quartile, third quartile).

b blood pressure of 140/90 mm Hg or higher, or antihypertensive medicine.

c average IMT of right and left common carotid artery.

CIMT was 0.099 mm (95%-confidence interval: 0.082–0.116) thicker in subjects with diabetes than in subjects with NGT or prediabetes in crude analysis. However, after adjustment for age and sex, there was no significant increase in subjects with diabetes compared to subjects with NGT/prediabetes (p = 0.17).

Among different groups of glucose regulation (i-IFG, i-IGT, IFG/IGT, previously undetected and previously known T2DM), significant increases in CIMT were seen compared to NGT in crude analyses (Table 2). Compared to subjects with NGT, CIMT was 0.086 mm (95%-CI: 0.061–0.112) thicker in subjects with IFG, and 0.081 mm (95%-CI: 0.064–0.098) thicker in subjects with IGT in crude analyses. After adjustment for age and sex, a significant increase was only seen in i-IFG compared to NGT. After additional adjustment for waist circumference, no increase was found among the categories of glucose regulation.

**Table 2:** Linear regression models with logarithm of carotid intima media thickness as dependent and glucose tolerance category as independent variable (e^ß^ (95%-CI))^a^.

	Model 1		Model 2		Model 3		Model 4	
	e^ß^ (95%-CI)	p	e^ß^ (95%-CI)	p	e^ß^ (95%-CI)	p	e^ß^ (95%-CI)	p
T2DM	1.12 (1.10–1.14)	<0.001	1.01 (0.996–1.03)	0.17	1.00 (0.99–1.01)	0.94	1.00 (0.99–1.02)	0.87
NGT or prediabetes	1 (ref)		1 (ref)		1 (ref)		1 (ref)	
known T2DM	1.14 (1.12–1.17)	<0.001	1.01 (0.99–1.03)	0.21	1.00 (0.98–1.02)	0.80	1.00 (0.98–1.02)	0.97
undetected T2DM	1.13 (1.10–1.17)	<0.001	1.02 (0.99–1.04)	0.17	1.00 (0.98–1.03)	0.74	1.00 (0.98–1.03)	0.80
IFG+IGT	1.11 (1.06–1.15)	<0.001	1.00 (0.97–1.03)	0.84	0.98 (0.95–1.01)	0.27	0.98 (0.95–1.01)	0.18
i-IGT	1.10 (1.08–1.12)	<0.001	1.01 (0.99–1.02)	0.29	1.00 (0.99–1.02)	0.93	1.00 (0.98–1.01)	0.88
i-IFG	1.11 (1.07–1.14)	<0.001	1.03 (1.002–1.05)	0.03	1.01 (0.99–1.04)	0.22	1.01 (0.99–1.03)	0.30
NGT	1 (ref)		1 (ref)		1 (ref)		1 (ref)	

CI: confidence interval; T2DM: type 2 diabetes; NGT: normal glucose tolerance; IFG: impaired fasting glucose; IGT: impaired glucose tolerance;

i-IFG: isolated impaired fasting glucose; i-IGT: isolated impaired glucose tolerance.

a For the interpretation of e^ß^ compare the methods section.

Model 1: crude model.

Model 2: adjusted for age and sex.

Model 3: adjusted for age, sex, and waist circumference.

Model 4: adjusted for age, sex, waist circumference, HDL cholesterol, LDL cholesterol, triglycerides, hypertension.

Overall, 2hPG and HbA1c were not significantly associated with CIMT after adjustment for age and sex (Table 3). FPG was significantly associated with CIMT after adjustment for age, sex, and hypertension (or age, sex, and cholesterol values), but not after adjustment for age, sex, and anthropometric measures.

**Table 3:** Linear regression models with logarithm of carotid intima media thickness as dependent variable and glycaemic measures as independent variables (e^ß^ (95%-CI))^a^.

	FPG^b^		2hPG^b^		HbA1c^c^	
Adjustment	e^ß^ (95%-CI)	p	e^ß^ (95%-CI)	p	e^ß^ (95%-CI)	p
age and sex	1.007 (1.002–1.011)	0.003	1.002 (0.999–1.004)	0.17	1.005 (0.997–1.013)	0.26
age, sex, and HT	1.005 (1.000–1.009)	0.04	1.001 (0.998–1.003)	0.65	1.001 (0.993–1.009)	0.78
age, sex, HDL, LDL	1.005 (1.000–1.009)	0.04	1.000 (0.998–1.002)	0.88	1.000 (0.992–1.008)	0.96
age, sex, and WC	1.002 (0.998–1.007)	0.30	0.999 (0.997–1.002)	0.51	0.998 (0.990–1.006)	0.55
age, sex, and BMI	1.003 (0.998–1.007)	0.25	0.999 (0.997–1.001)	0.48	0.999 (0.991–1.007)	0.72
full adjustment^d^	1.003 (0.998–1.008)	0.21	0.999 (0.996–1.001)	0.34	0.997 (0.989–1.005)	0.49

CI: confidence interval; FPG: fasting plasma glucose; 2hPG: 2-hour plasma glucose; HT: hypertension; HDL: high density lipoprotein.

cholesterol; LDL: low density lipoprotein cholesterol; WC: waist circumference; BMI: body mass index.

a For the interpretation of e^ß^ compare the methods section.

b per mmol/l.

c per %.

d adjusted for age, sex, waist circumference, HDL cholesterol, LDL cholesterol, triglycerides, hypertension.

In a fully adjusted logistic regression model, age, sex, hypertension, waist circumference, HDL and LDL cholesterol were all independently associated with elevated CIMT whereas the association was significant neither for HbA1c nor for FPG nor for 2hPG (Table 4).

**Table 4:** Risk factors of carotid intima media thickness (≥75^th^ percentile): Multivariate logistic regression model.

Risk factor	OR (95%-CI)
Age (per year)	1.12 (1.11–1.14)*
Sex (male versus female)	1.44 (1.13–1.82)*
Hypertension (yes versus no)	1.44 (1.14–1.81)*
HDL cholesterol (per mmol/l)	0.64 (0.45–0.90)*
LDL cholesterol (per mmol/l)	1.35 (1.20–1.52)*
Waist circumference (per cm)	1.02 (1.01–1.03)*
Fasting plasma glucose (per mmol/l)^a^	1.03 (0.94–1.14)

OR: odds ratio; CI: confidence interval; HDL: high density lipoprotein; LDL:

low density lipoprotein.

* p<0.05.

a When fasting plasma glucose was exchanged for 2-hour plasma glucose.

and for HbA1c, respectively, ORs (95%-CI) were as follows:

2-hour plasma glucose (per mmol/l): 0.98 (0.92–1.03).

HbA1c (per %): 1.10 (0.92–1.31).

When adjusted linear regression models as shown in Tables 2 and 3 were fitted separately for men and women, a lack of association between blood glucose and CIMT was seen in both sexes. In age-adjusted models, CIMT was neither elevated in men with diabetes (e^ß^ = 1.01 (95%-CI: 0.99–1.04)) nor in women with diabetes (e^ß^ = 1.01 (95%-CI: 0.98–1.03)) compared to men and women without diabetes, respectively. Furthermore, in age-adjusted models, none of the categories of glucose regulation was associated with CIMT in men and women with the exception of IFG and CIMT in women (e^ß^ = 1.04 (95%-CI: 1.004–1.08) (reference: NGT)). After further adjustment for waist circumference, the latter association was no longer significant in women (e^ß^ = 1.03 (95%-CI: 0.99–1.07)).

After adjustment for age and waist circumference, associations between fasting glucose, and 2-hour plasma glucose, respectively, with CIMT were no longer significant (p = 0.15, and p = 0.16, respectively, in men; p = 0.67, and p = 0.50, respectively, in women). Age-adjusted associations between HbA1c and CIMT were not significant neither in men nor in women (p = 0.20, and p = 0.93, respectively).

In fully adjusted logistic regression models, none of the three measures of glycaemia was associated with elevated CIMT when analyses were confined to subjects with NGT or to subjects with diabetes (data not shown). Moreover, results shown in Table 4 were similar when subjects with previously known diabetes were excluded, or when analyses were confined to subjects aged ≥50 years. Exclusion of persons with former myocardial infarction or stroke did not alter results of Tables 2, 3, and 4.

## Discussion

The main result of this population-based study is that associations between hyperglycaemia and CIMT which were found in crude analyses disappeared after adjustment for age, sex, and anthropometric variables. Contrary to measures of hyperglycaemia, traditional cardiovascular risk factors like BMI, waist circumference, hypertension, and dyslipidaemia, were all significantly associated with CIMT.

At first sight, this is a surprising result because there may be a causal link between glucose and atherosclerosis through various mechanisms like increased oxidative stress or nonenzymatic glycosylation of proteins and lipids [22]. Nevertheless, it must be seen that (A) literature findings about the association of glucose levels and CIMT are more inconclusive than it may appear in the light of published meta-analyses, and that (B) the role of hyperglycaemia for the development of atherosclerotic diseases may often be overestimated.

### (A) Inconclusive Findings in Former Studies on Hyperglycaemia and CIMT

When Faeh et al. included BMI in multiple linear regression models the relationship between diabetes and CIMT was borderline significant (p: 0.05–0.09), and it disappeared after inclusion of waist circumference instead of BMI [16]. However, in the ARIC study, CIMT was significantly larger in type 2 diabetes after adjusting for BMI, cholesterol, and hypertension [18]. An association of IFG with CIMT was not found in adjusted analyses [16], [23]. Larger CIMT values in persons with IGT compared to NGT were not reported after matching for BMI and waist-to-hip ratio [4]. In a prospective study, IGT did not predict CIMT levels after adjustment for CVD risk factors and BMI [15].

Table S1 in the supplementary material gives an overview of studies concerning the association of glycaemic measures and CIMT. From this table, it can be seen that significant associations between fasting glucose and CIMT were not seen in multivariate analyses [24]–[28], and that results were quite ambiguous regarding the association between 2-hour glucose, and HbA1c, respectively, with CIMT [17], [25], [29]–[34].

### (B) The Role of Hyperglycaemia for the Development of Atherosclerosis

Various literature findings support the idea that glucose is a risk factor for atherosclerosis, but possibly of minor importance than traditional CVD risk factors. In a systematic review, categories of pre-diabetes were only modestly associated with CVD risk: For IFG (110 to 125 mg/dl), the relative risk for eight studies was 1.12 (95%-CI: 1.00 to 1.25) compared to NGT after adjustment for age, smoking, blood pressure and lipids; for IGT, the summary estimate was 1.20 (95%-CI: 1.06 to 1.35) in six studies with adjustment for the same variables [35].

Stern et al. developed a model for the prediction of cardiovascular diseases which included age, sex, ethnicity, lipids, blood pressure, BMI, family history and smoking as traditional CVD risk factors. Addition of 2-hour plasma glucose to this multivariate model led to an only marginal increase of the area under the receiver operating curve of 0.1% [36]. Accordingly, Meigs et al. found that fasting glucose was not an independent risk factor for CVD when it was added to a prediction model including sex and CVD risk factors [37]. 2-hour plasma glucose led to a significant, albeit only marginal improvement of diabetes prediction.

Chamnan et al. found that subjects with HbA1c <5.5% (<37 mmol/mol) and unfavourable cardiovascular risk profiles have a more than 10-fold larger risk of CVD than subjects with HbA1c of 6.0 to 6.4% (42 to 46 mmol/mol) and favourable cardiovascular risk profiles [38]. The authors did not question the role of hyperglycaemia as a CVD risk factor, but like our study these results demonstrate the great importance of traditional risk factors for atherosclerosis. This can also be seen from a recently published study with Chinese participants: male gender, age, BMI, hypertension, and LDL cholesterol were significant determinants of CIMT, whereas fasting glucose was not associated with CIMT in a multivariate regression model [39].

In the ARIC study, an association between a fasting glucose genetic risk score and IMT was found (p = 0.009) [40]. However, the difference for one standard deviation of the score was only 0.0048 mm and hence not clinically relevant, and, moreover, the analysis was adjusted for age, sex, and study centre, but not for metabolic risk factors. The authors did not exclude that the association between the genetic risk score and IMT might be due to nonglucose pathways.

### Limitations and Strengths

One limitation of our study is its cross-sectional design so that the temporality of associations between glucose levels and CIMT could not be assessed. Moreover, glucose levels were measured only once so that some individuals might be inaccurately diagnosed. Our study has several strengths. It is a population-based study with a large sample size, and CIMT has been measured according to the Rotterdam protocol. Several categorical and continuous measures of glycaemia were included in the analyses. To assess associations of glucose levels with CIMT, different sets of potential confounders were used. Finally, our findings were robust and were confirmed after changes of analysis strategies (for example, exclusion of previously known diabetes; sex and age stratification; using CIMT as a continuous and as a dichotomous variable).

### Conclusion

In this population-based study, traditional CVD risk factors, but none of the measures of hyperglycaemia were independently associated with CIMT. There is still a lack of studies where the relationship between blood glucose and CIMT is assessed with careful adjustment for risk factors, and there is a lack of prospective studies to see whether blood glucose levels predict CIMT. In a public health perspective, our results suggest that targeting subjects for the prevention of early atherosclerosis should not primarily be directed to categories of glucose like IFG and IGT, but take into account the whole spectrum of traditional CVD risk factors including glucose.

## Supporting Information

Table S1 Characteristics and results of studies on the association between glycaemic measures and carotid intima-media thickness (CIMT).
(DOCX)
