# Supplementary material for: Associations between Blood Glucose and Carotid Intima-Media Thickness Disappear after Adjustment for Shared Risk Factors: The KORA F4 Study
Source: PLoS One. 2012 Dec 21;7(12):e52590. doi: 10.1371/journal.pone.0052590 (PMC3528645; doi:10.1371/journal.pone.0052590)
Supplement: Table S1 — Characteristics and results of studies on the association between glycaemic measures and carotid intima-media thickness (CIMT). (DOCX) [file pone.0052590.s001.docx]

**Table S1.** Characteristics and results of studies on the association between glycaemic measures and carotid intima-media thickness (CIMT)

| **Study** | **Characteristics of study group** | **Glycaemic measure** | **Statistical method** | **adjusted for** | **Association with CIMT** |
| --- | --- | --- | --- | --- | --- |
| Einarson (2010) [2] | meta-analysis, 11 studies, n=15,592 | 2hPG | Pearson correlation | unadjusted | r=0.082 (0.066, 0.098) |
| Brohall (2006) [3] | meta-analysis, 23 studies, n=24,111 | Type 2 diabetes vs nondiabetic controls |  | unadjusted | difference in CIMT=0.134 mm (0.123-0.144) |
|  |  | IGT vs NGT |  | unadjusted | difference in CIMT=0.042 mm (0.014-0.071) |
| Brohall (2009) [4] | meta-analysis, 12 studies, n=5,787 | IGT vs NGT | t-test | age | difference in CIMT=0.030 mm (0.012-0.048) |
| Folsom (1994)[18] | n=7,956, women | T2DM vs FPG < 6.4mmol/l | MLR | age, race, smoking, artery depth, ARIC field center, BMI, HT, LDL, HLD | p < 0.01 |
|  |  | FPG 6.4 – 7.7 mmol/l vs FPG < 6.4 mmol/l | MLR | age, race, smoking, artery depth, ARIC field center, BMI, HT, LDL, HLD | P=0.32 |
|  | n=6,474, men | T2DM vs FPG < 6.4mmol/l | MLR | age, race, smoking, artery depth, ARIC field center, BMI, HT, LDL, HLD | p < 0.01 |
|  |  | FPG 6.4 – 7.7 mmol/l vs FPG < 6.4 mmol/l | MLR | age, race, smoking, artery depth, ARIC field center, BMI, HT, LDL, HLD | p =0.21 |
| Temelkova-Kurktschiev (1998) [24] | n=307, nondiabetic | FPG | Pearson correlation | unadjusted | p=0.007 |
|  |  |  | Pearson correlation | age, sex | n.s. |
| Temelkova-Kurktschiev (1999)[41] | n=142, newly detected type 2 DM | Newly detected T2DM (WHO 1999) vs NGT/i-IFG | t-test, Mann-Whitney U test | matched for age, sex | p<0.001 |
| Temelkova-Kurktschiev (2000) [27] | n=582 | FPG | Pearson / Spearman correlation | age, sex | r=0.10 (p=0.015) |
|  |  |  | MLR | age, sex, BP, BMI, WHR, TC, HDL, TG, biomarkers^#^ | n.s. |
|  |  | 2hPG | Pearson / Spearman correlation | age, sex | r=0.211 (p<0.001) |
|  |  |  | MLR | age, sex, BP, BMI, WHR, TC, HDL, TG, biomarkers^#^ | p<0.05 |
|  |  | HbA1c | Pearson / Spearman correlation | age, sex | r=0.123 (p=0.003) |
|  |  |  | MLR | age, sex, BP, BMI, WHR, TC, HDL, TG, biomarkers^#^ | n.s. |
| Hanefeld (1999a) [23] | N=208 | IFG vs controls (FPG < 6.1mmol/l) | Wilcoxon signed-rank test | matched for age, sex, BMI (separate analysis for subjects with and without IGT) | n.s. |
| Hanefeld (1999b) [25] | n=403, nondiabetic | FPG | Pearson correlation | age, sex | r=0.01 (n.s.) |
|  |  | 2hPG | Pearson correlation | age,sex | r=0.23 (p<0.001) |
|  |  | 2hPG | MLR | age, sex, TC, HDL, TG, C-peptide | p=0.007 |
| Goya (2003) [26] | N=125, type 2 DM | FPG | univariate linear regression | - | p=0.548 |
|  |  | HbA1c | univariate linear regression | - | p=0.620 |
| Selvin (2005) [34] | N=2060, with DM | HbA1c | Logistic regression | age, sex, race , WHR, LDL, HDL, HT, TG, smoking, alcohol | P < 0.05 in separate analyses for diagnosed and undiagnosed diabetes |
| Zhang (2006) [29] | n=160, nondiabetic | FPG | MLR | age, SBP, DBP, Triglycerides, TC, LDL, HDL, smoking, BMI, WC, ISI | n.s. |
|  |  | 2hPG | MLR | age, SBP, DBP, Triglycerides, TC, LDL, HDL, smoking, BMI, WC, ISI | p < 0.0001 |
| Faeh (2007) [16] | n=496 | i-IFG versus NGT | MLR | None / RF/ RF+BMI, RF+WC | n.s. |
|  |  | IFG/IGT versus NGT | MLR | age,sex | p<0.05 |
|  |  |  | MLR | age,sex, RF (LDL, HDL, SBP, smoking) | n.s. |
|  |  |  | MLR | age,sex, RF + BMI | n.s. |
|  |  |  | MLR | age,sex, RF + WC | n.s. |
|  |  |  | MLR | age,sex,RF + I | n.s. |
|  |  |  | MLR | age,sex,RF + BMI, WC, I | n.s. |
|  |  | Type 2 DM versus NGT | MLR | age,sex | p < 0.05 |
|  |  |  | MLR | age,sex, RF (LDL, HDL, SBP, smoking) | p: 0.05 – 0.09 |
|  |  |  | MLR | age,sex, RF + BMI | p: 0.05 – 0.09 |
|  |  |  | MLR | age,sex, RF + WC | n.s. |
|  |  |  | MLR | age,sex,RF + I | n.s. |
|  |  |  | MLR | age,sex,RF + BMI, WC, I | n.s. |
| Brohall (2009) [4] | 64-year-old women with IGT (n=205) or with NGT (n=195) | IGT vs NGT |  | partly matched for BMI and WHR | difference between IGT and NGT: 0.00 mm (-0.03, 0.03) |
| Huang (2011) [31] | n=1627, NGT | FPG | ANOVA | unadjusted | n.s. |
|  |  | 2hPG | ANOVA | unadjusted | p < 0.05 |
|  |  |  | MLR | age, sex,smoking, drinking status, BMI, SBP, DBP, TC, HDL, LDL, TG | p=0.79 |
|  |  | HbA1c | ANOVA | unadjusted | p<0.05 |
|  |  |  | MLR | age, sex,smoking, drinking status, BMI, SBP, DBP, TC, HDL, LDL, TG | p=0.016 |
| Bobbert (2010) [32] | n=1219, nondiabetic | FPG | MLR | unadjusted | n.s. |
|  |  | 2hPG | MLR | unadjusted | n.s. |
|  |  | HbA1c | Pearson correlation | unadjusted | r=0.31 (p<0.001) |
|  |  |  | MLR | age, sex, WC, smoking, SBP, HDL / TC ratio | p=0.002 |
| Hu (2010) [30] | n=474, type 2 DM | HbA1c | Logistic regression | age, WHR, diabetes duration, SBP, TG, HDL, LDL, PG60, 2hPG, PG180, PGS, UACglu | p=0.02 |
|  |  | 2hPG | Logistic regression | age, WHR, diabetes duration, SBP, TG, HDL, LDL, PG60, PG180, PGS, HbA1c, UACglu | p=0.001 |
| Zhu (2010) [33] | n=216, nondiabetic | HbA1c | Logistic regression | age,sex, HT, SBP, 2hPG | n.s. |
|  |  |  | Logistic regression | BMI, smoking, SBP, DBP, FPG, 2hPG, LDL, HDL | p=0.009 |
| Choi (2011) [17] | n=370, type 2 DM | HbA1c | ANCOVA | unadjusted | p=0.75 |
|  |  |  | ANCOVA | age, sex | p=0.50 |
|  |  |  | ANCOVA | age, sex, diabetes duration | p=0.32 |
|  |  |  | ANCOVA | age, sex, BMI, smoking, diabetes duration, HT, HDL, LDL, TG, FPG | p=0.12 |
| Fitch (2011) [28] | 54 healthy subjects | FPG | Pearson correlation | unadjusted | p =0.0005 |
|  |  |  | MLR | Age, sex, smoking, SBP, DBP,HbA1c, HDL, LDL, neck circumference | p=0.72 |
|  |  | 2hPG | Pearson correlation | unadjusted | p=0.18 |
| Bonora (2000) [15] | n=826, prospective study | IGT vs NGT | MLR | unadjusted | P=0.004 |
|  |  |  | MLR | age, sex | P=0.046 |
|  |  |  | MLR | age, sex, smoking, LDL, HDL, fibrinogen, HT, TG, BMI | P=0.103 |
|  |  | Type 2 DM vs NGT | MLR | unadjusted | p<0.001 |
|  |  |  | MLR | age, sex | p=0.024 |
|  |  |  | MLR | age, sex, smoking, LDL, HDL, fibrinogen, HT, TG, BMI | p=0.072 |

MLR: multiple linear regression; ANOVA: analysis of variance; ANCOVA: analysis of covariance

CIMT: carotid intima-media thickness; FPG: fasting plasma glucose; 2hPG: 2-hour plasma glucose; NGT: normal glucose tolerance; IFG: impaired fasting glucose; IGT: impaired glucose tolerance; T2DM: type 2 diabetes mellitus; BP: blood pressure; SBP: systolic blood pressure; DBP: diastolic blood pressure; HT: hypertension; BMI: body mass index; WHR: waist-to-hip ratio; WC: waist circumference; TC: total cholesterol; HDL: HDL cholesterol; LDL: LDL cholesterol; TG: triglycerides; ISI: insulin sensitivity index; RF: risk factors; I: serum fasting insulin; UACglu: under area curve of glucose; PG60: 1-hour plasma glucose; PG180: 3-hour plasma glucose; PGS: plasma glucose spikes

#von Willebrand factor, fibrinogen, plaminogen activator inhibitor (active), leucocytes, and albuminuria

Supplementary References

41. Temelkova-Kurktschiev T, Koehler C, Leonhardt W, Schaper F, Henkel E, et al. (1999) Increased intima-media thickness in newly detected type 2 diabetes. Diabetes Care 22: 333-338.
